# Dual-purpose extensor pollicis longus transfer for combined type I and IV rheumatoid thumb deformities

**DOI:** 10.1016/j.jpra.2025.11.035

**Published:** 2025-12-07

**Authors:** Sébastien Durand, Yves Harder, Jessica Billy, Thomas Orthmann

**Affiliations:** Department of Plastic, Reconstructive and Aesthetic Surgery and Hand Surgery, Centre Hospitalier Universitaire Vaudois (CHUV), Lausanne, Switzerland

**Keywords:** Boutonniere deformity, Thumb deformity, Rheumatoid arthritis, Extensor pollicis longus, Tendon transfer, Extensor pollicis brevis

## Abstract

The Nalebuff classification, widely used by hand surgeons, describes six different canonical patterns. However, combined deformities are rarely reported, despite their significant impact on surgical decision-making. We present a dual-purpose extensor pollicis longus (EPL) transfer designed to address combined type I Boutonnière deformity and type IV metacarpophalangeal (MP) joint ulnar instability in rheumatoid thumb deformities. In two cases, a transosseous tunnel was drilled from dorsal to ulnar at the base of the proximal phalanx of the thumb, allowing passage and dorsal fixation of the EPL tendon. The remaining EPL tendon was then anchored at the ulnar side of the metacarpal head to reconstruct the ulnar collateral ligament. This dual-purpose transfer restores active thumb extension and simultaneously stabilizes the MP joint, thereby eliminating compensatory adduction at the first carpometacarpal joint.

## Introduction

Despite medical therapy, thumb deformity frequently occurs in different ways over time in patients with established rheumatoid arthritis (RA) and significantly impairs hand function in general and thumb function in particular.^1^ Nalebuff’s classification differentiates rheumatoid thumb deformities into six common types,^2^ among which types I and IV are of particular interest in this report.

Type 1, also known as the Boutonnière deformity, is the most common pathology of the thumb ray, occurring in around 60 % to 80 % of all long-term RA patients.^3^ Boutonniere deformity is caused by chronic synovitis of the MP joint, which progressively overstretches the joint capsule and leads to elongation of the extensor pollicis brevis (EPB), eventually resulting in the characteristic deformity involving MP joint flexion and IP joint hyperextension.

Type IV however, also known as the “gamekeeper’s thumb,” results from overstretching or rupture of the ulnar collateral ligament of the MP joint. The thumb becomes radially deviated, and the carpometacarpal (CMC) joint is secondarily positioned in adduction with shortening of the first dorsal interosseous and adductor muscles. An association between type I and IV is therefore logical, as both share the same origin, i.e., chronic synovitis of the MP joint.

In case of reducible deformity with no major cartilaginous involvement visible on plain radiographs, Nalebuff proposed a surgical correction for type 1 deformity using EPL rerouting.^2^ Indeed, the EPL tendon is transected distally and fixed with the EPB tendon to the base of the proximal phalanx of the thumb. Extension of the interphalangeal (IP) joint is then achieved by expansion of the intrinsic muscles still in continuity with the remaining distal segment of the EPL tendon. Later, authors, including Iwamoto^4^ and Oda^5^ reported limitations concerning this procedure, prompting modifications like rerouting without transection or adductor release to reduce recurrence.

We present a modified surgical approach using the EPL transfer, specifically designed for combined type I and IV deformities according to Nalebuff. This technique, illustrated through two clinical cases, aims to simultaneously correct the Boutonnière deformity, correct ulnar instability, while preserving MP joint function and optimizing first web space opening.

## Case report

### Patient 1

A 31-year-old woman with RA affecting both thumbs was treated for a combined thumb deformity according to Nalebuff type I and IV on the right side.

A longitudinal dorsal incision was made over the MP joint. We visualized an EPL tendon inserted to the distal phalanx and an EPB tendon that ran distally along the EPL as a conjoint tendon, ending at the IP joint. Direct traction on the EPB produced IP joint extension.

**Figure 1:**
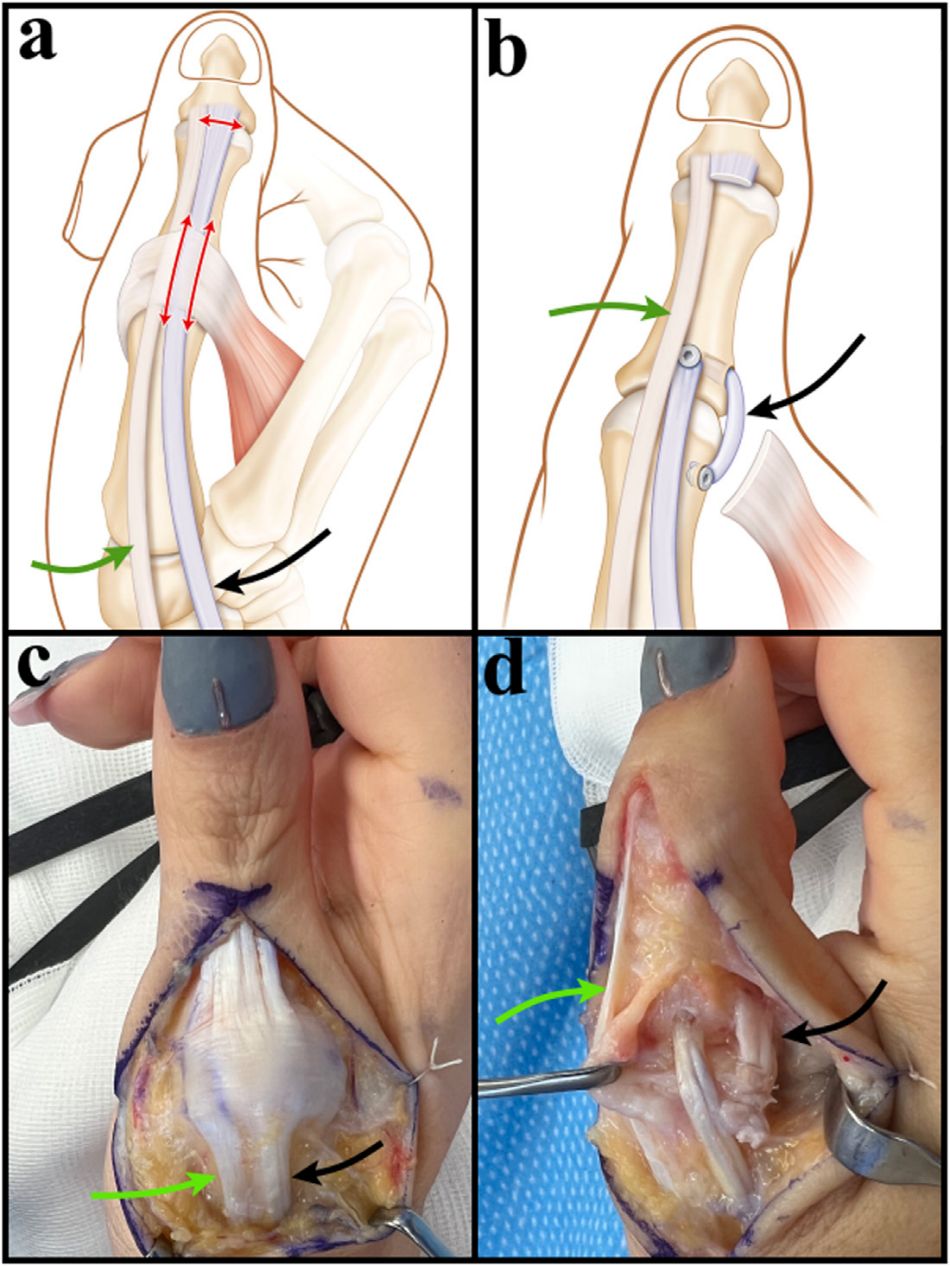
Schematic and intraoperative illustration of the dual-purpose EPL tendon transfer (patient 1). The EPL tendon was split longitudinally (a) and pulled through the transosseous tunnel from dorsal to ulnar. Then, the remaining EPL tendon was anchored to the ulnar side of the metacarpal head to reconstruct the ulnar collateral ligament (b, d). The EPB tendon (green arrow) runs distally along the EPL tendon (black arrow), like a conjoint tendon and ends at the level of the thumb’s IP joint (c, d).

The EPL was split longitudinally between the EPB and ulnar dorsal aponeurosis and was divided distally over the IP joint. Synovial tissue around the MP joint was excised. Thereafter, a 2.0 mm transosseous tunnel was drilled from dorsal to ulnar at the base of the thumb’s first phalanx (Figure 1), through which the EPL tendon was pulled through. Tension was adjusted by defining the EPL tendon’s length to hold the MP joint in 10° flexion, and the tendon was finally secured with a 2.5 mm interference screw (Arthrex, Naples, FL) through the dorsal drill hole. The remaining EPL tendon was anchored to the ulnar side of the metacarpal head of the first ray to reconstruct the ulnar collateral ligament. Dorsal ulnar aponeurosis was then sutured to the EPB tendon.

Postoperatively, the MP joint was splinted in ∼10° flexion with the IP joint left free. After 4 weeks, the splint was removed, and mobilization was initiated under occupational therapy guidance, continuing for 2 months.

**Figure 2:**
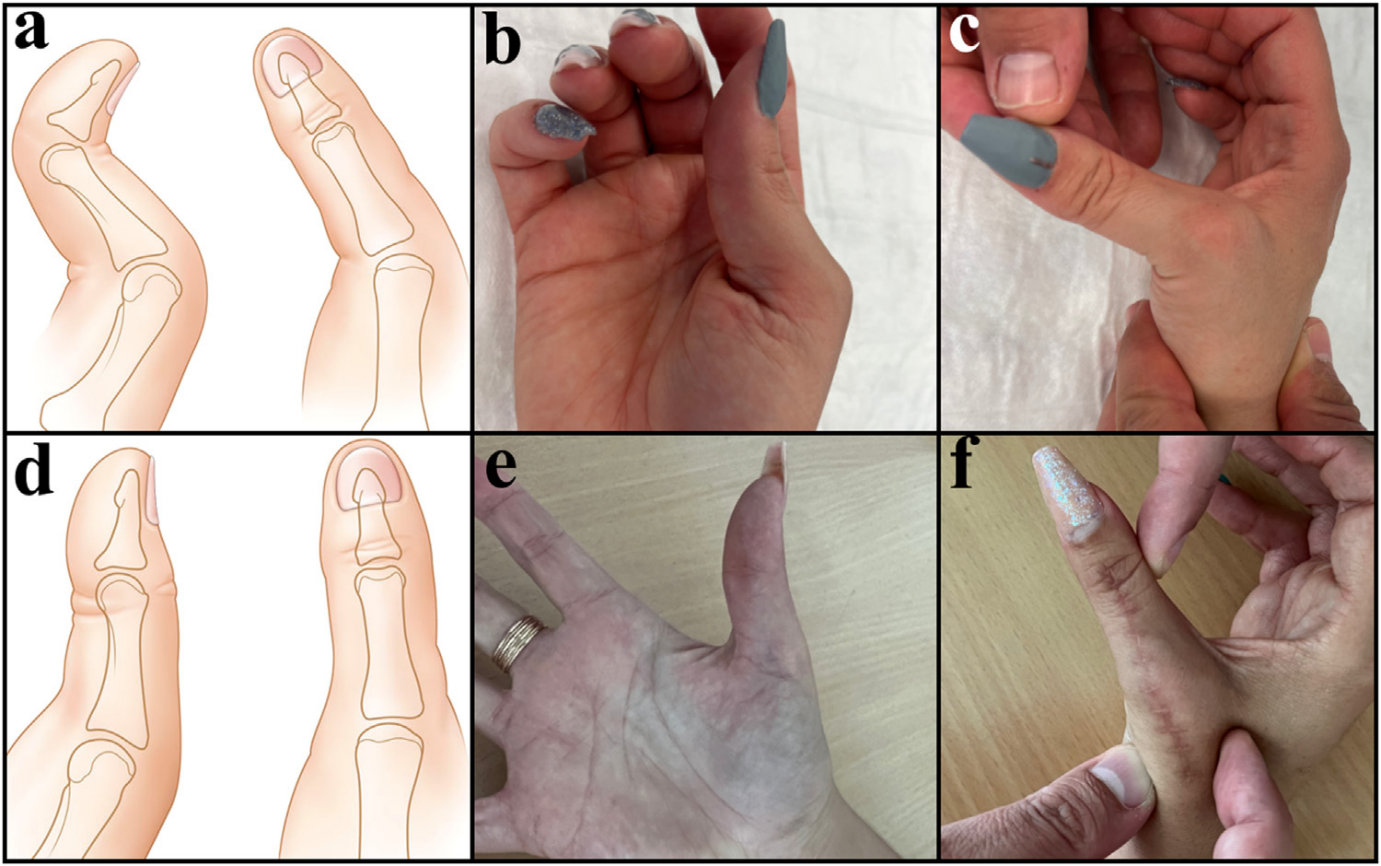
Patient 1 with combined type I (a, b) and type IV (a, c) deformities. 12 months after EPL tendon transfer, no MP joint instability was observed, with good opening of the first web space. Normalized range of motion was observed in the MP and IP joints (d, e, f).

At 12-month follow-up, the patient was asymptomatic, with restored MP joint stability and satisfactory widening of the first web space. Pinch strength improved to 8 kg from 5 kg preoperatively. MP and IP joints’ active ranges of motion improved respectively to 0/0/50° (from 0/40/50°) and 20/0/60° (from 20/0/0°). Complete IP joint range of motion was preserved due to the distal EPB insertion on the distal phalanx (Figure 2).

### Patient 2

A 41-year-old woman with RA affecting both thumbs presented with a combined type I and IV deformity on the right side, associated with dorsoradial IP joint dislocation and radiographic evidence of osteoarthritis predominantly involving the IP joint.

**Figure 3:**
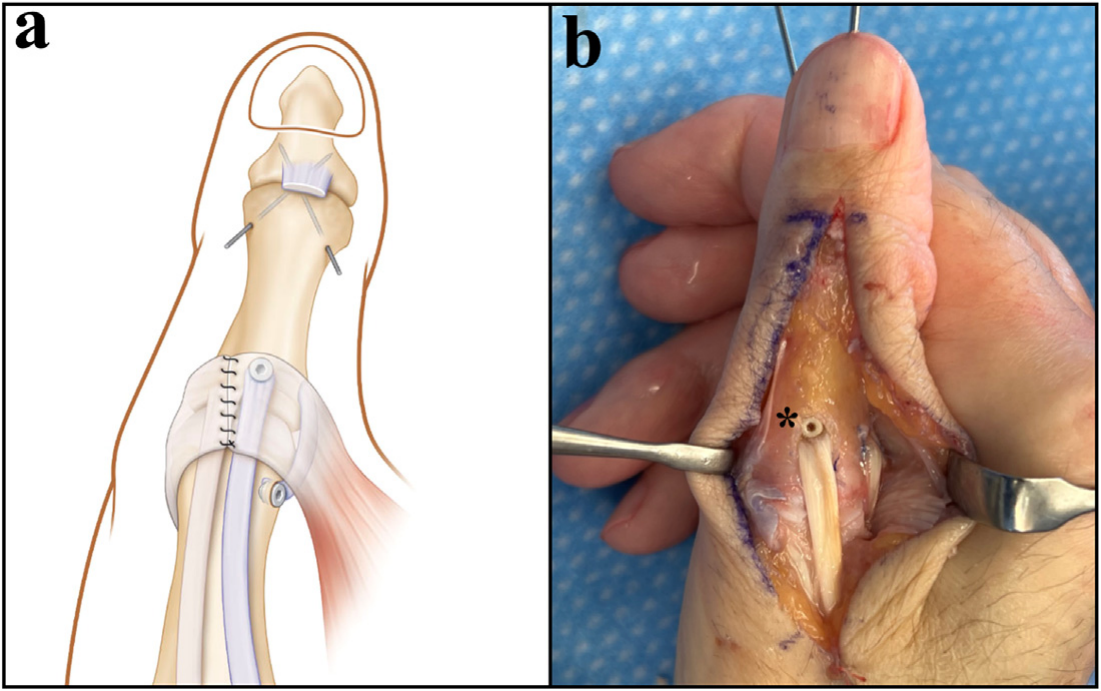
Schematic and intraoperative illustration of the dual-purpose EPL tendon transfer with IP joint arthrodesis (patient 2). EPL tendon was split longitudinally and pulled through the transosseous tunnel from dorsal to ulnar and secured with a 2.5 mm interference screw (*). The remaining EPL tendon was anchored to the ulnar side of the metacarpal head to reconstruct the ulnar collateral ligament.

The surgical approach followed the same principles as in Patient 1, except that an IP joint arthrodesis was performed due to advanced degenerative changes. The EPL tendon was rerouted and anchored as described previously to restore MP joint stability and extension (Figure 3).

**Figure 4:**
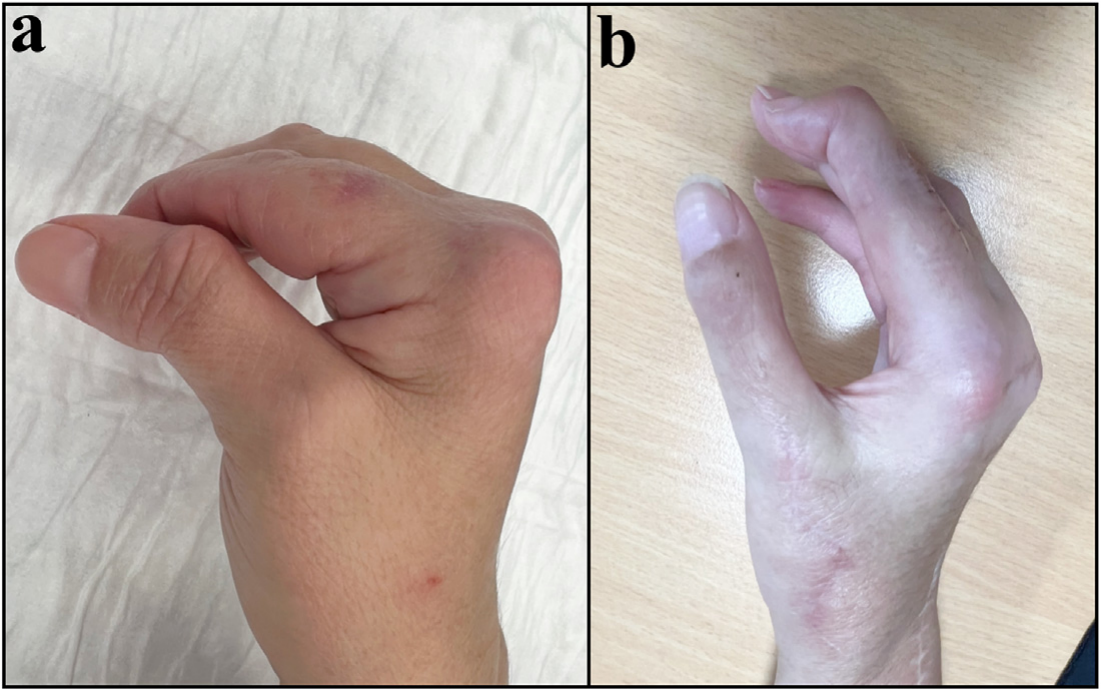
Patient 2 with combined deformities and dorsoradial dislocation of the thumb’s IP joint (a). 8 months after EPL tendon transfer, no MP joint instability was noted, with good opening of the first web space (b). Active MP extension was restored, and range of motion was 10/0/30°.

At 8-month of follow-up, the patient was asymptomatic, stability at the MP joint was restored and the opening of the first web space was satisfactory (Figure 4). The pinch force was 5 kg (compared with 1 kg before surgery) and the MP joint’s active range of motion was 10/0/30° (from 0/0/30° before surgery).

## Discussion

The surgical technique proposed herein has been inspired by the rerouting technique of the EPL tendon in Boutonnière deformity^2^ and by the combined procedure involving reconstruction of the ulnar collateral ligament of the MP joint of the thumb together with restoration of thumb opposition, as previously described by Su et al.^6^ in cases of thumb hypoplasia. This latter technique uses the flexor digitorum superficialis (FDS) tendon of the ring finger, which is fixed to the bone and to the periosteum on the radial side of the thumb’s metacarpal head. The tendon is then pulled through a bony tunnel or drill hole from radial to ulnar, with the remaining FDS tendon secured at the ulnar side of the base of the proximal phalanx to reconstruct the ulnar collateral ligament.

Though anatomical variants of the EPB and EPL tendons are common and therefore clinically relevant in rheumatoid thumb surgery. Okita et al. examined 103 cadaveric thumbs and compared their findings with surgical records from 23 patients who underwent surgical reconstruction for rheumatoid thumb Boutonnière deformity. The EPB tendon was consistently present in these patients. Insertion of the EPB tendon at the distal phalanx of the thumb was significantly more frequent in patients undergoing surgery for rheumatoid thumb Boutonnière deformity compared to the general population (64 % vs 29 %). When the EPB tendon inserts at the distal phalanx, it lacks bony attachment around the MP joint,^7^ potentially predisposing to Boutonnière deformity due to synovitis and swelling at the MP joint. In such cases, the EPB muscle may develop a conjoint tendon with the EPL muscle, allowing the EPL tendon to be distally divided if needed (patient 1). This anatomical variation was found to be advantageous in the first case, allowing preservation of the IP joint motion without need for arthrodesis. In contrast, in the second case, advanced IP joint degeneration required fusion; therefore, the transfer could be performed without concern for EPB anatomical variations, as its function was no longer required. These two situations underline both the versatility and the limitations of the proposed technique, which depend on joint integrity and the presence of suitable tendon anatomy.

As authors^2^^,^^8^ have highlighted, other patterns of thumb deformities beyond the six classical types do exist. Accordingly, their identification is crucial to ensure tailored treatment. This technique offers therefore a potential alternative to arthrodesis when no cartilage damage is present at the MP joint in combined Nalebuff type I and IV thumb deformities while simultaneously correcting both instability and deformity.

## Ethical approval declaration

Our Institution (CHUV-Centre Hospitalier Universitaire Vaudois) does not require ethical approval for reporting case series (<or =5).

## Informed consent declaration

Written informed consent was obtained from the patient for their anonymized information to be published in this article.

## Author contribution

Sébastien Durand: Conceptualization, Methodology, writing – review & editing. Yves Harder: writing – review & editing. Jessica Billy: writing and reviewing. Thomas Orthmann: writing and reviewing.

## Declaration of generative AI and AI-assisted technologies in the writing process

This scientific manuscript has been primarily written by human authors, with the involvement of AI-generated technologies only for the purpose of shortening the text. While Generative Artificial Intelligence (AI) and AI-assisted technologies have been utilized to streamline the text and enhance its clarity, the substantive content, conceptualization, and interpretation have been exclusively carried out by human researchers.

## Funding

None.

## Declaration of competing interest

None declared.
